# Joint and Independent Effects of Alcohol Drinking and Tobacco Smoking on Oral Cancer: A Large Case-Control Study

**DOI:** 10.1371/journal.pone.0068132

**Published:** 2013-07-10

**Authors:** José Leopoldo Ferreira Antunes, Tatiana Natasha Toporcov, Maria Gabriela Haye Biazevic, Antonio Fernando Boing, Crispian Scully, Stefano Petti

**Affiliations:** 1 School of Public Health, University of São Paulo, São Paulo, SP, Brazil; 2 School of Dentistry, University of São Paulo, São Paulo, SP, Brazil; 3 Post-Graduate Program of Public Health, Federal University of Santa Catarina, Brazil, Florianópolis, SC, Brazil; 4 Professor Emeritus, University College London, London, United Kingdom; 5 Department of Public Health and Infectious Diseases, Sapienza University, Rome, Italy; The University of Texas M. D. Anderson Cancer Center, United States of America

## Abstract

Alcohol drinking and tobacco smoking are assumed to have significant independent and joint effects on oral cancer (OC) development. This assumption is based on consistent reports from observational studies, which, however, overestimated the independent effects of smoking and drinking, because they did not account for the interaction effect in multivariable analyses. This case-control study sought to investigate the independent and the joint effects of smoking and drinking on OC in a homogeneous sample of adults. Case patients (N = 1,144) were affected by invasive oral/oropharyngeal squamous cell carcinoma confirmed histologically, diagnosed between 1998 and 2008 in four hospitals of São Paulo (Brazil). Control patients (N = 1,661) were not affected by drinking-, smoking-associated diseases, cancers, upper aero-digestive tract diseases. Cumulative tobacco and alcohol consumptions were assessed anamnestically. Patients were categorized into never/ever users and never/level-1/level-2 users, according to the median consumption level in controls. The effects of smoking and drinking on OC adjusted for age, gender, schooling level were assessed using logistic regression analysis; Model-1 did not account for the smoking-drinking interaction; Model-2 accounted for this interaction and included the resultant interaction terms. The models were compared using the likelihood ratio test. According to Model-1, the adjusted odds ratios (ORs) for smoking, drinking, smoking-drinking were 3.50 (95% confidence interval –95CI, 2.76–4.44), 3.60 (95CI, 2.86–4.53), 12.60 (95CI, 7.89–20.13), respectively. According to Model-2 these figures were 1.41 (95CI, 1.02–1.96), 0.78 (95CI, 0.48–1.27), 8.16 (95CI, 2.09–31.78). Analogous results were obtained using three levels of exposure to smoking and drinking. Model-2 showed statistically significant better goodness-of-fit statistics than Model-1. Drinking was not independently associated with OC, while the independent effect of smoking was lower than expected, suggesting that observational studies should be revised adequately accounting for the smoking-drinking interaction. OC control policies should focus on addictive behaviours rather than on single lifestyle risk factors.

## Introduction

Tobacco smoking and alcohol drinking are lifestyle risk factors which play an etiological role in oral cancer development with sufficient evidence. Such a high level of evidence is corroborated by a multitude of consistent observational studies published since the 70′s, which reported that these lifestyle risk factors were significantly associated with oral cancer [1]–[4]. Subsequent observational studies reported that oral cancer risk in subjects exposed to both smoking and drinking was greater than additive. More specifically, it was higher than the sum of the two individual risks attributable to smoking alone and to drinking alone. Such smoking-drinking joint (or interaction) effect was assessed informally, splitting samples into strata according to various levels of exposure to smoking and drinking and reporting the differences in oral cancer risk estimates between different strata [3], [5]. Subsequently, pooled- and meta-analyses were designed to formally estimate the magnitude of this joint effect: two studies used the Multiplicative Interaction Parameter and found that the joint effect was three [6] and two [7] times greater than the individual effects of smoking and drinking multiplied by each other; another study investigated the Interaction Contrast Ratio and found that the joint effect was greater than the additive effect of both exposures by a factor of two [8].

These data demonstrated that the smoking-drinking interaction has a statistically and clinically significant effect on oral cancer. Therefore, observational studies designed to investigate the effects of smoking and drinking on oral cancer must necessarily account for this significant joint effect, because the absent account of the interaction term leads to overestimate the independent effects of smoking and drinking on oral cancer, in addition to insufficient adjustment for distal covariates in studies assessing other risk factors. The simplest method to account for such a joint effect in regression analysis is the interaction term approach, which implies that an additional variable -the interaction term- is included in the model [9], [10].

Astonishingly, no observational study published during the last twenty-five years has accounted for the smoking-drinking interaction (reviewed by [3]–[5], [8]). These considerations raise the suspect that the independent effects of smoking and drinking on oral cancer are currently overestimated, because meta-analyses and systematic reviews are based on these observational studies [1]–[4].

This study aimed to contribute to this discussion, by assessing the hypothesis that when the statistical model properly accounts for the smoking-drinking joint effect, the independent effects of smoking and drinking on oral cancer risk are lower than expected. Specifically, we designed a case-control study to compare the conventional method to assess the individual effects of tobacco smoking and alcohol drinking which does not account for the interaction effect, with an alternative assessment that accounts for such a joint effect of both exposures.

## Methods

### Ethics Statement

All the four study centres, involved in this case-control study, observed Brazilian and international statutes on ethics in research regarding human beings; all patients signed an informed consent and ethical clearance was given by the Research Ethics Committees of all participating hospitals. More specifically, the “Comitê de Ética em Pesquisa do Complexo Hospitalar Heliópolis”, the “Comitê de Ética em Pesquisa - CEP - do Hospital A.C.Camargo”, the “Comitê de Ética em Pesquisa em Seres Humanos do Instituto do Câncer Arnaldo Vieira de Carvalho” and the “Comissão de Ética para Análise de Projetos de Pesquisa da Diretoria Clínica do Hospital das Clínicas da Faculdade de Medicina da Universidade de São Paulo”.

### Study Design

We designed a hospital-based, case-control study including patients with squamous cell carcinoma of the oral cavity and oropharynx confirmed histologically.

It was necessary to control or adjust the effects of smoking and drinking for the effects of other factors. For diseases like oral cancer with complex multifactorial aetiology, many behavioural, genetic, environmental factors are even unknown [11], [12]. Therefore, it is impossible to build an observational study which accounts for all these factors. In addition, studies which do not account for hidden/non-investigated/unknown variables overestimate the effects of the remaining investigated factors. We overcame this serious problem including demographic variables (age, gender and socio-economic level) in the set of covariates and, most importantly, selecting cases and controls from the same underlying population, that is, patients who sought for care in four hospitals of São Paulo (Brazil), who followed similar referral routes, that is, the general practitioners. The important advantage of selecting the sample from a homogeneous population is that in these circumstances, the hidden/non-investigated/unknown factors are part of the background environment, are assumed to be uniformly distributed and, hence, can be disregarded, thus also simplifying the statistical methods [13], [14]. Therefore, single-centre studies account for unidentified factors better than multi-centre studies.

In addition, in order to increase the chance that non-significant associations between the investigated variables and oral cancer were truly due to lack of associations rather than to high level of beta error, we collected the largest possible sample. We, therefore, did not pre-assess the minimum sample size, but we assessed the statistical power of the study on the basis of the characteristics of the final sample.

Finally, since the two principal exposure variables were evaluated anamnaestically, we assessed exposure to smoking and drinking using methods endorsed and promoted by the International Agency for Research on Cancer (IARC).

### Sample Selection

#### Cases

From November 1998 to December 2008 we enrolled patients undergoing treatment for oral and oropharyngeal cancer in four main hospitals of the city of São Paulo, Brazil (Hospital Heliópolis; Hospital das Clínicas; Hospital A.C. Camargo; Instituto do Câncer Arnaldo Vieira de Carvalho) to participate in case-control studies assessing different aetiological hypotheses. One of these four studies [15] assessed patients with head and neck cancer enrolled for broader multicentre studies (namely, the “International study of environment, viruses and cancer of the oral cavity and the larynx”, Latin-American section, and the “Clinical Genome of Cancer Project”) [16]. The remaining studies [17]–[19] exclusively assessed patients with oral and oropharyngeal cancer.

The current sample was gathered by merging the databases from the four hospitals. The case group was exclusively composed by newly diagnosed patients with invasive oral (C01–C06, International Classification of Diseases, 10^th^ revision) and oropharyngeal (C09–C10) squamous cell carcinoma, confirmed histologically. More specifically, cancers of tongue (C01, C02), gum (C03), floor of mouth (C04), palate (C05), other unspecified parts of mouth (C06), tonsil (C09) and oropharynx (C10) were included, while cancers of lip (C00), nasopharynx (C11), hypopharynx (C13) and other sites of lip, oral cavity and pharynx (C14) were excluded. Stage-0 cancers, corresponding to carcinoma in situ, were excluded because they were not necessarily invasive.

In order to restrict cases to those who had the same reasonable possibility of having had their disease induced by the exposure under investigation [13], only patients from hospitals of the same town were selected, a condition which guaranteed that unidentified or non-investigated oral cancer risk factors were uniformly distributed within the population from which cases and controls were selected.

#### Controls

Control patients were individuals assisted in outpatient units of the same hospitals who followed the same referral routes as case patients (i.e., primary healthcare services, mainly general practitioners and general dental practitioners). Controls were not to be affected by diseases potentially related with drinking and smoking exposures. In addition, subjects with current or past experience of cancer or aero-digestive tract diseases were not eligible.

The data from the four study centres, merged in this study, followed the matching procedures for gender and age. As one of the study centres [15] focused on the broad category of head and neck cancer, it was necessary to select only patients with oral and oropharyngeal cancer from the database of that centre and to exclude patients with cancers of nasopharynx, hypopharynx and larynx, while all the controls were considered. For this reason, in the present study the control group was larger than the case group and an adjustment for age and gender also was mandatory.

### Explanatory Variables

Specifically trained examiners interviewed participants immediately after their clinical consultation in a separate room; hospital files were consulted to register information comprised in the medical record of patients. All cases and controls underwent identical personal interviews regarding gender, age, schooling level, tobacco smoking and alcohol drinking.

The assessment of smoking and drinking habits followed the methods endorsed by IARC [3], [4], [20], validated, standardized and extensively used in broad epidemiologic studies within the INHANCE Consortium (see, for example, [6], [21], [22]). More specifically, the questionnaire considered sequential patterns of frequency, duration and type of product consumed during the subject’s lifetime. Patients reporting not having smoked at least one daily cigarette during a whole year were considered never smokers. A cigar was considered equivalent to four cigarettes, and each pipe serve equivalent to three cigarettes [4]. Cumulative doses of tobacco exposure were calculated in terms of pack-years (one pack-year equals to one package of cigarettes smoked daily for one year). Two schemes of classification were used: dichotomous categorisation (ever smokers, never smokers) and three categories (never smokers, level-1 smokers and level-2 smokers, according to the median of pack-years reported by controls).

Patients reporting having never consumed at least one drink at a regular monthly basis were considered non-drinkers. Alcohol drinking was measured by grams of ethanol, considering that one litre of ethanol weighs 798 g and that beer contains 5% ethanol in volume; wine 12%; liqueurs 30% and distilled spirits 41% [20]. Cumulative exposure to alcohol was expressed in gram-years (grams of ethanol consumed daily multiplied by the number of drinking years). Two schemes of classification were used: dichotomous categorisation (ever drinkers, never drinkers) and three categories (never drinkers, level-one drinkers and level-two drinkers, according to the median of gram-years reported by controls).

### Statistical Analysis

The differences between cases and controls regarding age, gender and schooling level were assessed through logistic regression analysis using controls as reference group. Age was categorized into <45, 45–49, 50–54, 55–59, 60–64, 65–69, >69 years, while schooling level into <5, 5–8, >8 years of formal education. Variables which provided odds ratios (ORs) for oral cancer which were statistically significant at 95% level were included in the statistical models as confounders.

In order to explore the level of beta error, the power of the study, corresponding to “1-β”, was assessed with the formula, “Z_β_ = {√ N [r/(r+1)] (p_1_–p_2_)^2^ [1/p(1-p)]} - Z_α_”, where “Z_β_” is the value of the standard normal distribution corresponding to the value of β; “N” is the sample size (cases+controls); “r” is the ratio of controls to cases; “p_1_” and “p_2_” are the proportions of exposed to drinking in cases and controls; “p” is the average proportion of exposed to drinking “Z_α_” is the value of the standard normal distribution corresponding to the value of α = 0.05 using a two-sided test (i.e., 3.92).

The effect of drinking and smoking on oral cancer risk was explored through unadjusted ORs with 95% confidence intervals (95% CI), which were assessed by unconditional logistic regression [23].

Unconditional logistic regression analysis was also used to assess the adjusted individual and joint effects of smoking and drinking on oral cancer risk. Age, gender and schooling level were considered as potential confounders if they resulted significantly associated with oral cancer. Two analyses were run. In the first analysis, the exposure variables drinking and smoking were treated as binary (never, reference group; ever, risk group). In the second analysis, they were categorized into three levels (never, reference group; level-1 and level-2, risk groups). Two models were designed:

*Model-1* was the simplest model, conventionally used by observational studies investigating the effects of lifestyle risk factors on cancer. This model accounted exclusively for confounding and assumed that: (1) smoking and drinking exerted individual effects on oral cancer risk; (2) they were reciprocally associated; (3) they did not exert interaction effect.

According to this model, the OR for the “ever smoking, ever drinking” category was obtained by the inverse of the logarithm of the sum of the coefficients of smoking and drinking, as provided by logistic regression.

*Model-2* was the model proposed here to investigate the effects of lifestyle risk factors. This model accounted for confounding and interaction and assumed that: (1) smoking and drinking exerted individual effects on oral cancer risk; (2) they were reciprocally associated; (3) they exerted interaction effect.

In order to account for the interaction effect, the interaction term approach was preferred to other methods, such as the stratified analysis with joint categories, because it was the most widely applicable and practical [24]. As already noted, this approach implied the use of the smoking-drinking interaction term, given by the product between the two exposure variables. When they were treated as binary, there was only one interaction term, with score 1 for the “ever smoking, ever drinking” category and score 0 for the remaining categories (i.e., “never smoking, never drinking”, “never smoking, ever drinking”, “ever smoking, never drinking”). The OR for the “ever smoking, ever drinking” category was obtained by the inverse of the logarithm of the sum of the coefficients of smoking, drinking and smoking-drinking interaction term.

When exposure variables were categorized into three levels, four interaction terms were generated, one for every type of joint exposure, namely, “level-1 smoking, level-1 drinking”, “level-1 smoking, level-2 drinking”, “level-2 smoking, level-1 drinking”, “level-2 smoking, level-2 drinking”.

In order to check whether the OR estimates were artificially inflated or overestimated by excess collinearity and multicollinearity, before running the regression analyses, the explanatory variables were tested by means of pairwise Pearson’s correlation coefficient (r) and variance inflation factor (VIF). The highest acceptable values for r and VIF were set at 0.6 and 10, respectively. The robustness of the OR estimates was also investigated through validation analysis. Namely, the case and the control groups were split into two halves: two random variables were generated, one for cases, one for controls; the case and the control groups were ordered according to these variables; the first half of cases and the first half of controls were grouped in sub-sample 1, the remainder in sub-sample 2. Coefficients for smoking, drinking and interaction terms and goodness of fit of regression models were re-estimated in both sub-samples. According to the validation analysis, coefficients, compared using the 95% CIs, must not differ between sub-samples, while p-values of −2log likelihood must be similar [9], [25].

The goodness of fit of the regression models was assessed using the −2log likelihood (the lower, the better fit) and Pseudo-R^2^ (the higher, the better fit). The goodness-of-fit of Model-1 and Model-2 was statistically compared using the likelihood ratio test, with approximately χ^2^ distribution. The smoking-drinking interaction effect was considered statistically significant if the goodness-of-fit was significantly better in Model-2 than in Model-1.

All analyses were performed using Stata 12.0 (Stata Corporation, College Station, Texas, US, 2011).

## Results

The study included 1,144 cases and 1,661 controls (**Table 1**). Only few subjects, less than ten per group, did not provide their informed consent to study participation, some of them because of speech impairment, due to their status, some of them because they declared they had no time for the interview.

**Table 1:** Characteristics of case and control patients and unadjusted odds ratios.

Subjects	Cases n (%)	Controls n (%)	OR (95% CI)
Total	1,144	1,661	
Gender			
Female	221 (19.3)	445 (26.8)	1.00
Male	923 (80.7)	1,216 (73.2)	1.53 (1.27–1.83)
Age (years)			
<45	141 (12.3)	324 (19.5)	1.00
45–49	168 (14.7)	236 (14.2)	1.64 (1.24–2.16)
50–54	228 (19.9)	241 (14.5)	2.17 (1.66–2.84)
55–59	197 (17.2)	241 (14.5)	1.88 (1.43–2.47)
60–64	151 (13.2)	203 (12.2)	1.71 (1.28–2.28)
65–69	106 (9.3)	187 (11.3)	1.30 (0.96–1.78)
>69	153 (13.4)	229 (13.8)	1.54 (1.16–2.04)
Schooling level (years of formal education)			
<5	398 (34.8)	607 (36.5)	1.00
5–8	550 (48.1)	662 (39.9)	1.27 (1.06–1.50)
>8	196 (17.1)	392 (23.6)	0.76 (0.62–0.94)

São Paulo, Brazil, 1998–2008.

The distribution of cases and controls by gender, age group and schooling level is displayed in **Table 1**. The unadjusted ORs for oral and oropharyngeal cancer were significantly higher among males, the elderly and individuals with less than 8 years of formal education. Gender, age and schooling level explained 2.1% of the variance of regression residuals, which reinforces the need of including these covariates as confounders in the regression models fitting behavioural exposures and oral cancer.

**Table 2** describes the distribution of cases and controls according to categories of exposure to smoking and drinking. The median cumulative tobacco consumption among control patients was 28 pack-years, while the median cumulative alcohol consumption was 862 gram-years. These values were the thresholds used to split smoking and drinking exposures into level-1 (moderate consumption) and level-2 (heavy consumption). Using the data from this Table, the estimated values of Z_β_ were 20.71 and 21.74 for smoking and drinking, respectively, which resulted in power levels higher than 99.9% for both smoking and drinking (data not in Table). **Table 2** also describes the unadjusted assessment of associations between the outcome and these covariates. The highest risk estimates were reported for level-2 smokers (OR, 7.43; 95% CI 5.94–9.30), level-2 drinkers (OR, 6.73; 95% CI 5.35–7.91) and ever smokers-ever drinkers (OR, 5.85; 95% CI 4.59–7.46).

**Table 2:** Distribution of cases and controls according to tobacco smoking and alcohol drinking and unadjusted odds ratios.

Category	Cases n (%)	Controls n (%)	OR (95% CI)
Median cumulativeconsumption			
Tobacco smoking(pack-years)	39.3	28.0	
Alcohol drinking(gram-years)	2,058.4	862.0	
Smoking status			
Never smoker	121 (10.6)	620 (37.3)	1.00
Ever smoker	1,023 (89.4)	1,041 (62.7)	5.04 (4.07–6.23)
Level-1 smoker^a^	269 (23.5)	521 (31.4)	2.65 (2.07–3.38)
Level-2 smoker^a^	754 (65.9)	520 (31.4)	7.43 (5.94–9.30)
Drinking status			
Never drinker	199 (17.4)	769 (46.3)	1.00
Ever drinker	945 (82.6)	906 (53.7)	4.21 (3.50–5.06)
Level-1 drinker^b^	194 (17.0)	446 (26.9)	1.68 (1.34–2.11)
Level-2 drinker^b^	751 (65.6)	446 (26.9)	6.73 (5.35–7.91)
Smoking and drinking status			
Never smoker and never drinker	96 (8.4)	427 (25.7)	1.00
Never smoker and ever drinker	25 (2.2)	193 (11.6)	0.58 (0.36–0.92)
Ever smoker and never drinker	103 (9.0)	342 (20.6)	1.34 (0.98–1.83)
Ever smoker and ever drinker	920 (80.4)	699 (42.1)	5.85 (4.59–7.46)

a Cumulative consumption: level-1 smoker ≤28 pack-years; level-2 smoker >28 pack-years.

b Cumulative consumption: level-1 drinker ≤862 gram-years; level-2 drinker >862 gram-years.

In the multivariable assessment (**Table 3**), Model-1 registered that the two lifestyle exposures, considered both independently and jointly, were highly and significantly associated with oral cancer. The ORs estimated by Model-2 were generally lower than those estimated by Model-1 and the independent effect of alcohol drinking resulted no longer associated with the disease (OR, 0.78; 95% CI, 0.48–1.27). Notwithstanding this observation, the goodness of fit of Model-2 was significantly better than the goodness of fit of Model-1 (p<0.001) at the likelihood ratio test.

**Table 3:** Individual and joint effects of smoking and drinking on oral and oropharyngeal cancer adjusted for gender, age, schooling level.

Category	Model-1 OR (95% CI)	Model-2 OR (95% CI)
Exposures dichotomously classified^a^		
Ever smoker	3.50 (2.76–4.44)	1.41 (1.02–1.96)
Ever drinker	3.60 (2.86–4.53)	0.78 (0.48–1.27)
Ever smoker and ever drinker	12.60 (7.89–20.13)	8.16 (2.09–31.78)
Exposures classified in three categories^b^		
Never smoker and never drinker	1.00	1.00
Never smoker and level-1 drinker	1.68 (1.29–2.20)	0.63 (0.40–1.00)
Never smoker and level-2 drinker	5.71 (4.41–7.39)	1.51 (0.88–2.57)
Level-1 smoker and never drinker	2.06 (1.57–2.70)	1.17 (0.80–1.71)
Level-1 smoker and level-1 drinker	3.47 (2.03–5.94)	2.42 (0.57–10.30)
Level-1 smoker and level-2 drinker	11.78 (6.94–19.97)	8.61 (2.05–36.13)
Level-2 smoker and never drinker	4.61 (3.53–6.01)	2.05 (1.39–3.03)
Level-2 smoker and level-1 drinker	7.76 (4.56–13.21)	6.32 (1.61–24.83)
Level-2 smoker and level-2 drinker	26.32 (15.59–44.42)	19.10 (3.85–94.72)

Model-1 did not account for the smoking-drinking interaction, smoking and drinking were therefore treated only as confounders. Model-2 accounted for the smoking-drinking interaction.

a Model-1: Pseudo-R^2^ = 0.118; −2log likelihood = 3346.826. Model-2: Pseudo-R^2^ = 0.134; −2log likelihood = 3285.967.

Difference in goodness of fit between Model-1 and Model-2: likelihood ratio test χ^2^ = 60.859, p<0.001.

b Model-1: Pseudo-R^2^ = 0.175; −2log likelihood = 3130.079. Model-2: Pseudo-R^2^ = 0.186; −2log likelihood = 3088.889.

Difference in goodness of fit between Model-1 and Model-2: likelihood ratio test χ^2^ = 41.190, p<0.001.

An analogous result was obtained when both exposures were classified using three categories (**Table 3**). Model-1 showed that all the smoking-drinking categories different from the reference group had a direct and statistically significant association with the disease. However, according to Model-2, no significant association was observed between the disease and the various smoking-drinking categories for individuals who smoked less than 28 pack-years (level-1 smokers) and drank less than 682 gram-years (level-1 drinkers). The ORs for all the categories of concurrent exposure to smoking and drinking estimated by Model-2 were lower than the ORs estimated by Model-1. Nevertheless, at the likelihood ratio test, Model-2 had a significantly better fit than Model-1 (p<0.001).

The values of “r” and VIF were below the limits of 0.6 and 10, suggesting that the coefficient estimates were substantially not inflated or overestimated. According to the validation analysis, the coefficient estimates and goodness of fit of regression models did not change substantially, thus corroborating the robustness of the various risk estimates (data not in Table).

## Discussion

The most important findings of this study were that (1)the independent effect of drinking substantially decreased and was no longer associated with oral and oropharyngeal cancer accounting for the smoking-drinking interaction term; (2)the independent effect of smoking also considerably decreased, although it remained significantly associated with the disease; (3)the smoking-drinking joint effect remained significantly associated with oral cancer; (4)regression models accounting for the smoking-drinking interaction term had a significantly better fit than those exclusively assessing the individual effects of behavioural exposures.

We gathered an exceptionally high number of participants for a single case-control study on oral cancer risk factors (see previous observational studies reviewed by [3], [8], [26], [27]), an option which considerably increased the power of the study (higher than 99% for both smoking and drinking exposures), that is, the chance that non-significant associations between explanatory and outcome variables were due to lack of associations between behavioural risk factors and oral cancer. In addition, cases and controls belonged to the same homogeneous study population, a situation which helps control for other risk factors for oral cancer, because in these circumstances the unidentified and non-investigated factors are part of the background environment and can be disregarded. This is an important advantage for the investigation of diseases with complex multifactorial aetiology like oral cancer [13], [14]. Therefore, large size and homogeneity of sample increased the internal validity of this study, that is, the reliability of the reported risk estimates [28].

Being hospital-based, this study was potentially subjected to criticism regarding selection bias, since the present sample was not representative of all residents in the city of São Paulo. Indeed, factors which bring people to public hospitals, such as financial standings, area of residence, ethnicity, religious affiliation, are not distributed uniformly within the underlying study population, thus making it difficult to define the population from which our case patients arose. In order to minimize the consequences of selection bias on the consistency of results, we decided to select hospital-based controls who followed the same referral route as cases. In these circumstances, the underlying study population was the same in the two groups and was identifiable in those residents in the city of São Paulo who sought for care in public hospitals [13], [14], [23]. In addition, the selection of case and control patients from the same underlying study population minimized the degree of information bias, since hospital-based controls tend to show the same levels of cooperativeness and accuracy in providing information as the hospital-based cases, thus reducing the potential differences between these two groups in the quality of recall of past exposures [13].

Information bias regarding lifestyle variables, which are generally underreported by heavy users is another important form of bias [29]–[31]. Since reliability of lifestyle variables is particularly low when they are treated quantitatively, we classified lifestyle variables into categories, considering that treating variables semi-quantitatively would reduce the negative effect of information bias on the reliability of risk estimates [32], [33]. This approach is also preferred by experts in alcohol drinking epidemiology, who make international comparisons using qualitative data [32].

Recall bias and interviewer bias, two specific forms of information bias, are also relevant limitations of case-control studies [13], [23], [34]. Patients diagnosed with oral and oropharyngeal cancer may have spent some time pondering on deleterious habits that may have contributed to the disease. Therefore, cases would be more likely to recall alcohol drinking and tobacco smoking than controls. This limitation is difficult to overcome in the scope of a case-control study. Interviewer bias also could not be excluded, as interviewers were trained, but not blinded. Recall and interviewer biases may have resulted in overestimates of smoking and drinking exposure among cases. Consequently, the smoking and drinking risk estimates might have been artificially higher than the true risks, which would be even lower if these forms of bias were completely controlled.

Finally, it is possible that other variables, strongly associated with both drinking and smoking, also were associated with oral and oropharyngeal cancer risk. Indeed, smoking and drinking are not only associated with each other, they are also associated with other behavioural risk factors for cancer and other degenerative diseases, such as unsafe sex, use of other addictive substances, unhealthy diet, low physical exercise, etc. [35], [36]. The problem beyond these apparently different forms of substance-use and behavioural addiction lies in the individuals’ personality, as early initiation of risk behaviours is associated with other risk-taking behaviours [2], [37]. Therefore, the joint exposure to smoking and drinking may also imply a potentially etiological role of hidden variables.

The data of the present study were partly corroborated. Indeed, two multi-centre studies from IARC, one based on seventeen centres from Europe and America [6] and another based on fourteen centres from Europe [7] reported that 40% oral cancer cases were attributable to the smoking-drinking joint effect and that the independent effect of drinking was non-significant. However, both studies reported that smoking alone was responsible for approximately 20% oral cancer cases. As noted, multi-centre studies are not homogeneous and, therefore, tend to overestimate the effects of the investigated variables because they do not account for unidentified factors [13].

This study may have implications in the design of effective oral cancer control policy. Indeed, policies based on the control of a single risk factor are fated to fail in the long term, as already demonstrated for alcohol drinking [38], due to the addictive behaviour of individuals, who are likely to re-start smoking and/or drinking or to quit smoking and/or drinking but start with another unsafe lifestyle, such as cigars instead of cigarettes, unsafe diet instead of alcohol, tobacco chewing instead of tobacco smoking, etc. (see, for example, [39]–[41]). Therefore, our study demonstrated that the exposure to a single lifestyle is not only uncommon, as previously demonstrated, but it also does not pose an important risk for oral cancer. Conversely, multiple exposures are very frequent, due to the widespread addictive behaviour, and are a serious risk for oral cancer.

In conclusion, the present data suggest that in this large-sized and homogeneous sample from São Paulo, drinking was not independently associated with oral/oropharyngeal cancer, while the independent effect of smoking was lower than expected. Instead, the joint effect of drinking and smoking was significantly associated with this condition. We strongly recommend that observational studies from other countries are designed accounting for such an important interaction, thus allowing investigating whether this result may be generalized to populations with different lifestyles, and with various drinking and smoking modalities. According to these findings, oral cancer control policies should focus primarily on the addictive behaviour which induces people to adopt several unsafe lifestyles.

## References

[1] Argiris A, Karamouzis MV, Raben D, Ferris RL (2008) Head and neck cancer. Lancet 371 (9625): 1695–1709. 10.1016/S0140-6736(08)60728-X PMC7720415 18486742

[2] Petti S (2009) Lifestyle risk factors for oral cancer. Oral Oncol 45 (4–5): 340–350. 10.1016/j.oraloncology.2008.05.018 18674956

[3] IARC Working Group on the Evaluation of Carcinogenic Risks to Humans (2010) Alcohol Consumption and Ethyl Carbamate. IARC Monogr Eval Carcinog Risks Hum 96: 3–1383. 21735939 PMC4781168

[4] IARC Working Group on the Evaluation of Carcinogenic Risks to Humans (2004) Tobacco smoke and involuntary smoking. IARC Monogr Eval Carcinog Risks Hum 83: 1–1438. 15285078 PMC4781536

[5] Petti S, Masood M, Messano GA, Scully C (2013) Alcohol is not a risk factor for oral cancer in nonsmoking, betel quid non-chewing individuals. A meta-analysis update. Ann Ig 25 (1): 3–14. 10.7416/ai.2013.1901 23435775

[6] Hashibe M, Brennan P, Chuang SC, Boccia S, Castellsague X, et al (2009) Interaction between tobacco and alcohol use and the risk of head and neck cancer: pooled analysis in the International Head and Neck Cancer Epidemiology Consortium. Cancer Epidemiol Biomarkers Prev 18 (2): 541–550. 10.1158/1055-9965.EPI-08-0347 PMC3051410 19190158

[7] Anantharaman D, Marron M, Lagiou P, Samoli E, Ahrens W, et al (2011) Population attributable risk of tobacco and alcohol for upper aerodigestive tract cancer. Oral Oncol 47 (8): 725–731. 10.1016/j.oraloncology.2011.05.004 21684805

[8] Petti S, Mohd M, Scully C (2012) Revisiting the association between alcohol drinking and oral cancer in nonsmoking and betel quid non-chewing individuals. Cancer Epidemiol 36 (1): e1–e6. 10.1016/j.canep.2011.09.009 22015229

[9] Bagley SC, White H, Golomb BA (2001) Logistic regression in the medical literature: Standards for use and reporting with particular attention to one medical domain. J Clin Epidemiol 54 (10): 979–985. 10.1016/s0895-4356(01)00372-9 11576808

[10] Marill KA (2004) Advanced statistics: linear regression, part II: multiple linear regression. Acad Emerg Med 11 (1): 94–102. 10.1197/j.aem.2003.09.006 14709437

[11] Warnakulasuriya S (2009) Global epidemiology of oral and oropharyngeal cancer. Oral Oncol 45 (4–5): 309–316. 10.1016/j.oraloncology.2008.06.002 18804401

[12] Scully C (2011) Oral cancer aetiopathogenesis; past, present and future aspects. Med Oral Patol Oral Cir Bucal 16 (3): e306–e311. 10.4317/medoral.16.e306 21441876

[13] dos Santos Silva I (1999) Cancer Epidemiology: Principles and Methods. Lyon: International Agency for Research on Cancer.

[14] Schoenbach VJ, Rosamond VD (2000) Multicausality. Effect modification. In: Schoenbach VJ, Rosamond VD eds. Understanding the Fundamental of Epidemiology – An Evolving Text. Chapel Hill: University of North Carolina, 381–422.

[15] Boing AF, Antunes JL, de Carvalho MB, de Góis Filho JF, Kowalski LP, et al (2011) How much do smoking and alcohol consumption explain socioeconomic inequalities in head and neck cancer risk? J Epidemiol Community Health 65 (8): 709–714. 10.1136/jech.2009.097691 20724282

[16] Wünsch-Filho V, Eluf-Neto J, Lotufo PA, Silva WA Jr, Zago MA (2006) Epidemiological studies in the information and genomics era: experience of the clinical Genome of cancer project in São Paulo, Brazil. Braz J Med Biol Res 39 (4): 545–553. 10.1590/s0100-879x2006000400016 16612479

[17] Toporcov TN, Antunes JLF, Tavares MR (2004) Fat food habitual intake and risk of oral cancer. Oral Oncol 40 (9): 925–931. 10.1016/j.oraloncology.2004.04.007 15380171

[18] Biazevic MG, Toporcov TN, Antunes JL, Rotundo LD, Brasileiro RS, et al (2011) Cumulative coffee consumption and reduced risk of oral and oropharyngeal cancer. Nutr Cancer 63 (3): 350–356. 10.1080/01635581.2011.536065 21462087

[19] Toporcov TN, Tavares GE, Rotundo LDB, Biazevic MGH, Vaccarezza GF, et al (2012) Do tobacco and alcohol modify protective effects of diet on oral carcinogenesis? Nutr Cancer 64 (8): 1182–1189. 10.1080/01635581.2012.721155 23163847

[20] IARC Working Group on the Evaluation of Carcinogenic Risks to Humans (1988) Alcohol drinking. IARC Monogr Eval Carcinog Risks Hum 44: 1–378. 3236394 PMC6421508

[21] Purdue MP, Hashibe M, Berthiller J, La Vecchia C, Dal Maso L, et al (2009) Type of alcoholic beverage and risk of head and neck cancer–a pooled analysis within the INHANCE Consortium. Am J Epidemiol 169 (2): 132–142. 10.1093/aje/kwn306 PMC2727255 19064644

[22] Lubin JH, Gaudet MM, Olshan AF, Kelsey K, Boffetta P, et al (2010) Body mass index, cigarette smoking, and alcohol consumption and cancers of the oral cavity, pharynx, and larynx: modeling odds ratios in pooled case-control data. Am J Epidemiol 171 (12): 1250–1261. 10.1093/aje/kwq088 PMC2915496 20494999

[23] Schlesselman JJ (1982) Case-control studies: design, conduct, analysis. New York: Oxford University Press.

[24] Szklo M, Nieto FJ (2007) Stratification and Adjustment: Multivariate Analysis in Epidemiology. In: Szklo M, Nieto FJ eds. Epidemiology: beyond the basics -2^nd^ ed. Sudbury: Jones and Barlett Publishers, 227–298.

[25] Petti S, Scully C (2007) Oral cancer knowledge and awareness: primary and secondary effects of an information leaflet. Oral Oncol 43 (4): 408–415. 10.1016/j.oraloncology.2006.04.010 16996779

[26] Turati F, Garavello W, Tramacere I, Bagnardi V, Rota M, et al (2010) A meta-analysis of alcohol drinking and oral and pharyngeal cancers. Part 2: results by subsites. Oral Oncol 46 (10): 720–726. 10.1016/j.oraloncology.2010.07.010 20728401

[27] Tramacere I, Negri E, Bagnardi V, Garavello W, Rota M, et al (2010) A meta-analysis of alcohol drinking and oral and pharyngeal cancers. Part 1: Overall results and dose-risk relation. Oral Oncol 46 (7): 497–503. 10.1016/j.oraloncology.2010.03.024 20444641

[28] Petti S (2009) Why guidelines for early childhood caries prevention could be ineffective amongst children at high risk. J Dent 38 (12): 946–955. 10.1016/j.jdent.2010.09.002 20837088

[29] Greenfield TK, Kerr WC (2008) Alcohol measurement methodology in epidemiology: recent advances and opportunities. Addiction 103 (7): 1082–1099. 10.1111/j.1360-0443.2008.02197.x PMC2782942 18422826

[30] Gorber SC, Schofield-Hurwitz S, Hardt Jvasseur G, Tremblay M (2009) The accuracy of self-reported smoking: a systematic review of the relationship between self-reported and cotinine-assessed smoking status. Nicotine Tob Res 11 (1): 12–24. 10.1093/ntr/ntn010 19246437

[31] Metcalfe C, Macleod J, Smith JD, Hart CL (2008) The scope for biased recall of risk-factor exposure in case-control studies: evidence from a cohort study of Scottish men. Scand J Publ Health 36 (4): 442–445. 10.1177/1403494807088451 18539700

[32] Bloomfield K, Stockwell T, Gmel G, Rehn N (2003) International comparisons of alcohol consumption. Alcohol Res Health 27 (1): 95–109. PMC6676703 15301404

[33] Bekkering GE, Harris RJ, Thomas S, Mayer AM, Beynon R, et al (2008) How much of the data published in observational studies of the association between diet and prostate or bladder cancer is usable for meta-analysis? Am J Epidemiol 167 (9): 1017–1026. 10.1093/aje/kwn005 18403406

[34] Vandenbroucke JP, von Elm E, Altman DG, Gøtzsche PC, Mulrow CD, et al (2007) Strengthening the Reporting of Observational Studies in Epidemiology (STROBE): explanation and elaboration. PLoS Med 4 (10): e297. 10.1371/journal.pmed.0040297 PMC2020496 17941715

[35] Zacny JP (1990) Behavioral aspects of alcohol-tobacco interactions. Recent Dev Alcohol 8: 205–219. 2185518

[36] Schröder H, Elosua R, Marrugat J, REGICOR Investigators (2003) The relationship of physical activity with dietary cancer-protective nutrients and cancer-related biological and lifestyle factors. Eur J Cancer Prev 12 (4): 339–346. 10.1097/00008469-200308000-00016 12883389

[37] Grant JE, Potenza MN, Weinstein A, Gorelick DA (2010) Introduction to behavioural addictions. Am J Drug Alcohol Abuse 36 (5): 233–241. 10.3109/00952990.2010.491884 PMC3164585 20560821

[38] Room R, Babor T, Rehm J (2005) Alcohol and Public Health. Lancet 365 (9458): 519–530. 10.1016/S0140-6736(05)17870-2 15705462

[39] Grosshans M, Loeber S, Kiefer F (2011) Implications from addiction research towards the understanding and treatment of obesity. Addict Biol 16 (2): 189–198. 10.1111/j.1369-1600.2010.00300.x 21371174

[40] von der Goltz C, Kiefer F (2009) Learning and memory in the aetiopathogenesis of addiction: future implications for therapy? Eur Arch Psychiatry Clin Neurosci 259 Suppl 2S183–S187. 19876677 10.1007/s00406-009-0057-6

[41] Avena NM, Bocarsly ME, Hoebel BG, Gold MS (2011) Overlaps in the nosology of substance abuse and overeating: the translational implications of “food addiction”. Curr Drug Abuse Rev 4 (3): 133–139. 10.2174/1874473711104030133 21999687

